# Supplementary material for: Dynalign II: common secondary structure prediction for RNA homologs with domain insertions
Source: Nucleic Acids Res. 2014 Nov 21;42(22):13939–48. doi: 10.1093/nar/gku1172 (PMC4267632; doi:10.1093/nar/gku1172)
Supplement: SUPPLEMENTARY DATA [file supp_gku1172_nar-02021-z-2014-File012.zip › manual/javadoc/RNAstructure_java_interface/source/windows/ModuleWindow.html]

ModuleWindow


JavaScript is disabled on your browser.


- Overview
- Package
- Class
- Tree
- Index
- Help

- Prev Class
- Next Class

- Frames
- No Frames

- All Classes

- Summary:
- Nested |
- Field |
- Constr |
- Method

- Detail:
- Field |
- Constr |
- Method


RNAstructure\_java\_interface.source.windows

## Class ModuleWindow

- java.lang.Object
- - java.awt.Component
  - - java.awt.Container
    - - javax.swing.JComponent
      - - javax.swing.JInternalFrame
        - - RNAstructure\_java\_interface.source.windows.InternalWindow
          - - RNAstructure\_java\_interface.source.windows.ModuleWindow

- All Implemented Interfaces:
  :   ActionListener, ImageObserver, MenuContainer, Serializable, EventListener, Accessible, RootPaneContainer, WindowConstants

  Direct Known Subclasses:
  :   DynalignFoldWindow, DynalignRefoldWindow, Efn2Window, FoldDoubleWindow, FoldSingleWindow, FoldSuboptimalWindow, MaxExpectWindow, MultiWindow, OligoScreenWindow, OligoWalkWindow, PartitionDoubleWindow, PartitionSingleWindow, ProbKnotWindow, PseudoknotWindow, RefoldWindow, StochasticWindow

  ---

    

  ```
  public abstract class ModuleWindow
  extends InternalWindow
  ```

  A class responsible for creating and displaying a window that enables a
  user to input data for one of the RNAstructure modules.

  See Also:
  :   Serialized Form

- - ### Nested Class Summary

    - ### Nested classes/interfaces inherited from class javax.swing.JInternalFrame

      `JInternalFrame.AccessibleJInternalFrame, JInternalFrame.JDesktopIcon`
    - ### Nested classes/interfaces inherited from class javax.swing.JComponent

      `JComponent.AccessibleJComponent`
    - ### Nested classes/interfaces inherited from class java.awt.Container

      `Container.AccessibleAWTContainer`
    - ### Nested classes/interfaces inherited from class java.awt.Component

      `Component.AccessibleAWTComponent, Component.BaselineResizeBehavior, Component.BltBufferStrategy, Component.FlipBufferStrategy`
  - ### Field Summary

    Fields

    | Modifier and Type | Field and Description |
    | --- | --- |
    | `private GridBagConstraints` | `constraints` The GridBagConstraints for this window. |
    | `protected boolean` | `isRNA` A boolean, true if the calculation handles RNA, false if not. |
    | `private GridBagLayout` | `layout` The layout for this window. |
    | `protected JDialog` | `progressBar` The progress bar dialog for this window. |
    | `private static long` | `serialVersionUID` |

    - ### Fields inherited from class RNAstructure\_java\_interface.source.windows.InternalWindow

      `backend, dialogHandler, menuBar`
    - ### Fields inherited from class javax.swing.JInternalFrame

      `closable, CONTENT_PANE_PROPERTY, desktopIcon, FRAME_ICON_PROPERTY, frameIcon, GLASS_PANE_PROPERTY, iconable, IS_CLOSED_PROPERTY, IS_ICON_PROPERTY, IS_MAXIMUM_PROPERTY, IS_SELECTED_PROPERTY, isClosed, isIcon, isMaximum, isSelected, LAYERED_PANE_PROPERTY, maximizable, MENU_BAR_PROPERTY, resizable, ROOT_PANE_PROPERTY, rootPane, rootPaneCheckingEnabled, title, TITLE_PROPERTY`
    - ### Fields inherited from class javax.swing.JComponent

      `accessibleContext, listenerList, TOOL_TIP_TEXT_KEY, ui, UNDEFINED_CONDITION, WHEN_ANCESTOR_OF_FOCUSED_COMPONENT, WHEN_FOCUSED, WHEN_IN_FOCUSED_WINDOW`
    - ### Fields inherited from class java.awt.Component

      `BOTTOM_ALIGNMENT, CENTER_ALIGNMENT, LEFT_ALIGNMENT, RIGHT_ALIGNMENT, TOP_ALIGNMENT`
    - ### Fields inherited from interface javax.swing.WindowConstants

      `DISPOSE_ON_CLOSE, DO_NOTHING_ON_CLOSE, EXIT_ON_CLOSE, HIDE_ON_CLOSE`
    - ### Fields inherited from interface java.awt.image.ImageObserver

      `ABORT, ALLBITS, ERROR, FRAMEBITS, HEIGHT, PROPERTIES, SOMEBITS, WIDTH`
  - ### Constructor Summary

    Constructors

    | Modifier | Constructor and Description |
    | --- | --- |
    | `protected` | `ModuleWindow(String acid, String title)` Constructor. |
  - ### Method Summary

    Methods

    | Modifier and Type | Method and Description |
    | --- | --- |
    | `protected String` | `combineFileNames(String file1, String file2, String ext)` Combine two file names into a new file name. |
    | `protected void` | `doActions(String command)` Do any actions specific to this window. |
    | `protected abstract void` | `executeModuleAction(String command)` Execute an action within a particular module. |
    | `protected void` | `finishModule(String text)` Handle finishing of a module. |
    | `protected JComponent` | `getInputControl(Container hold, int index)` Get a particular input control from a given container. |
    | `protected JComponent` | `getInputControl(int index)` Get a particular input control from the content pane. |
    | `protected abstract void` | `makeInputControls()` Make any input controls necessary to get data from this window for a calculation. |
    | `protected void` | `makeStartButton(int x, int y)` Create the "START" button. |
    | `protected boolean` | `moduleInitialized(String result)` Check to see if the module was initialized correctly. |
    | `protected void` | `placeComponent(int x, int y, JComponent component)` . |
    | `protected String` | `replaceExtension(String file, String extension)` Replace the extension on a file name. |
    | `protected abstract void` | `runMainCalculation()` Run the module calculation in the back end. |
    | `protected void` | `setAnchorCenter()` Set the anchoring area for the GridBagConstraints to CENTER. |
    | `protected void` | `setAnchorNorth()` Set the anchoring area for the GridBagConstraints to NORTH. |
    | `protected void` | `setFillCenter()` Set the fill for the GridBagConstraints as CENTER. |
    | `protected void` | `setFillHorizontal()` Set the fill for the GridBagConstraints as HORIZONTAL. |
    | `protected void` | `setGrid(int width, int height)` Set the amount of space a component takes up in the grid. |
    | `protected void` | `setInsets(int top, int left, int bottom, int right)` Set the external padding of the constraints. |
    | `protected void` | `setPad(int xPad, int yPad)` Set the internal padding of the constraints. |
    | `private void` | `showProgressBar(boolean determinate)` Create a progress bar. |
    | `protected void` | `showProgressBarDeterminate()` Create a determinate progress bar. |
    | `protected void` | `showProgressBarIndeterminate()` Create an indeterminate progress bar. |

    - ### Methods inherited from class RNAstructure\_java\_interface.source.windows.InternalWindow

      `actionPerformed, setMenus, setTitles, viewWindow`
    - ### Methods inherited from class javax.swing.JInternalFrame

      `addImpl, addInternalFrameListener, createRootPane, dispose, doDefaultCloseAction, fireInternalFrameEvent, getAccessibleContext, getContentPane, getDefaultCloseOperation, getDesktopIcon, getDesktopPane, getFocusCycleRootAncestor, getFocusOwner, getFrameIcon, getGlassPane, getInternalFrameListeners, getJMenuBar, getLastCursor, getLayer, getLayeredPane, getMenuBar, getMostRecentFocusOwner, getNormalBounds, getRootPane, getTitle, getUI, getUIClassID, getWarningString, hide, isClosable, isClosed, isFocusCycleRoot, isIcon, isIconifiable, isMaximizable, isMaximum, isResizable, isRootPaneCheckingEnabled, isSelected, moveToBack, moveToFront, pack, paintComponent, paramString, remove, removeInternalFrameListener, reshape, restoreSubcomponentFocus, setClosable, setClosed, setContentPane, setCursor, setDefaultCloseOperation, setDesktopIcon, setFocusCycleRoot, setFrameIcon, setGlassPane, setIcon, setIconifiable, setJMenuBar, setLayer, setLayer, setLayeredPane, setLayout, setMaximizable, setMaximum, setMenuBar, setNormalBounds, setResizable, setRootPane, setRootPaneCheckingEnabled, setSelected, setTitle, setUI, show, toBack, toFront, updateUI`
    - ### Methods inherited from class javax.swing.JComponent

      `addAncestorListener, addNotify, addVetoableChangeListener, computeVisibleRect, contains, createToolTip, disable, enable, firePropertyChange, firePropertyChange, firePropertyChange, fireVetoableChange, getActionForKeyStroke, getActionMap, getAlignmentX, getAlignmentY, getAncestorListeners, getAutoscrolls, getBaseline, getBaselineResizeBehavior, getBorder, getBounds, getClientProperty, getComponentGraphics, getComponentPopupMenu, getConditionForKeyStroke, getDebugGraphicsOptions, getDefaultLocale, getFontMetrics, getGraphics, getHeight, getInheritsPopupMenu, getInputMap, getInputMap, getInputVerifier, getInsets, getInsets, getListeners, getLocation, getMaximumSize, getMinimumSize, getNextFocusableComponent, getPopupLocation, getPreferredSize, getRegisteredKeyStrokes, getSize, getToolTipLocation, getToolTipText, getToolTipText, getTopLevelAncestor, getTransferHandler, getVerifyInputWhenFocusTarget, getVetoableChangeListeners, getVisibleRect, getWidth, getX, getY, grabFocus, isDoubleBuffered, isLightweightComponent, isManagingFocus, isOpaque, isOptimizedDrawingEnabled, isPaintingForPrint, isPaintingOrigin, isPaintingTile, isRequestFocusEnabled, isValidateRoot, paint, paintBorder, paintChildren, paintImmediately, paintImmediately, print, printAll, printBorder, printChildren, printComponent, processComponentKeyEvent, processKeyBinding, processKeyEvent, processMouseEvent, processMouseMotionEvent, putClientProperty, registerKeyboardAction, registerKeyboardAction, removeAncestorListener, removeNotify, removeVetoableChangeListener, repaint, repaint, requestDefaultFocus, requestFocus, requestFocus, requestFocusInWindow, requestFocusInWindow, resetKeyboardActions, revalidate, scrollRectToVisible, setActionMap, setAlignmentX, setAlignmentY, setAutoscrolls, setBackground, setBorder, setComponentPopupMenu, setDebugGraphicsOptions, setDefaultLocale, setDoubleBuffered, setEnabled, setFocusTraversalKeys, setFont, setForeground, setInheritsPopupMenu, setInputMap, setInputVerifier, setMaximumSize, setMinimumSize, setNextFocusableComponent, setOpaque, setPreferredSize, setRequestFocusEnabled, setToolTipText, setTransferHandler, setUI, setVerifyInputWhenFocusTarget, setVisible, unregisterKeyboardAction, update`
    - ### Methods inherited from class java.awt.Container

      `add, add, add, add, add, addContainerListener, addPropertyChangeListener, addPropertyChangeListener, applyComponentOrientation, areFocusTraversalKeysSet, countComponents, deliverEvent, doLayout, findComponentAt, findComponentAt, getComponent, getComponentAt, getComponentAt, getComponentCount, getComponents, getComponentZOrder, getContainerListeners, getFocusTraversalKeys, getFocusTraversalPolicy, getLayout, getMousePosition, insets, invalidate, isAncestorOf, isFocusCycleRoot, isFocusTraversalPolicyProvider, isFocusTraversalPolicySet, layout, list, list, locate, minimumSize, paintComponents, preferredSize, printComponents, processContainerEvent, processEvent, remove, removeAll, removeContainerListener, setComponentZOrder, setFocusTraversalPolicy, setFocusTraversalPolicyProvider, transferFocusDownCycle, validate, validateTree`
    - ### Methods inherited from class java.awt.Component

      `action, add, addComponentListener, addFocusListener, addHierarchyBoundsListener, addHierarchyListener, addInputMethodListener, addKeyListener, addMouseListener, addMouseMotionListener, addMouseWheelListener, bounds, checkImage, checkImage, coalesceEvents, contains, createImage, createImage, createVolatileImage, createVolatileImage, disableEvents, dispatchEvent, enable, enableEvents, enableInputMethods, firePropertyChange, firePropertyChange, firePropertyChange, firePropertyChange, firePropertyChange, firePropertyChange, getBackground, getBounds, getColorModel, getComponentListeners, getComponentOrientation, getCursor, getDropTarget, getFocusListeners, getFocusTraversalKeysEnabled, getFont, getForeground, getGraphicsConfiguration, getHierarchyBoundsListeners, getHierarchyListeners, getIgnoreRepaint, getInputContext, getInputMethodListeners, getInputMethodRequests, getKeyListeners, getLocale, getLocation, getLocationOnScreen, getMouseListeners, getMouseMotionListeners, getMousePosition, getMouseWheelListeners, getName, getParent, getPeer, getPropertyChangeListeners, getPropertyChangeListeners, getSize, getToolkit, getTreeLock, gotFocus, handleEvent, hasFocus, imageUpdate, inside, isBackgroundSet, isCursorSet, isDisplayable, isEnabled, isFocusable, isFocusOwner, isFocusTraversable, isFontSet, isForegroundSet, isLightweight, isMaximumSizeSet, isMinimumSizeSet, isPreferredSizeSet, isShowing, isValid, isVisible, keyDown, keyUp, list, list, list, location, lostFocus, mouseDown, mouseDrag, mouseEnter, mouseExit, mouseMove, mouseUp, move, nextFocus, paintAll, postEvent, prepareImage, prepareImage, processComponentEvent, processFocusEvent, processHierarchyBoundsEvent, processHierarchyEvent, processInputMethodEvent, processMouseWheelEvent, remove, removeComponentListener, removeFocusListener, removeHierarchyBoundsListener, removeHierarchyListener, removeInputMethodListener, removeKeyListener, removeMouseListener, removeMouseMotionListener, removeMouseWheelListener, removePropertyChangeListener, removePropertyChangeListener, repaint, repaint, repaint, resize, resize, setBounds, setBounds, setComponentOrientation, setDropTarget, setFocusable, setFocusTraversalKeysEnabled, setIgnoreRepaint, setLocale, setLocation, setLocation, setName, setSize, setSize, show, size, toString, transferFocus, transferFocusBackward, transferFocusUpCycle`
    - ### Methods inherited from class java.lang.Object

      `clone, equals, finalize, getClass, hashCode, notify, notifyAll, wait, wait, wait`

- - ### Field Detail


    - #### constraints

      ```
      private GridBagConstraints constraints
      ```

      The GridBagConstraints for this window.


    - #### isRNA

      ```
      protected boolean isRNA
      ```

      A boolean, true if the calculation handles RNA, false if not.


    - #### layout

      ```
      private GridBagLayout layout
      ```

      The layout for this window.


    - #### progressBar

      ```
      protected final JDialog progressBar
      ```

      The progress bar dialog for this window.


    - #### serialVersionUID

      ```
      private static final long serialVersionUID
      ```

      See Also:
      :   Constant Field Values
  - ### Constructor Detail


    - #### ModuleWindow

      ```
      protected ModuleWindow(String acid,
                  String title)
      ```

      Constructor.

      Parameters:
      :   `acid` - The nucleic acid type, as a string.
      :   `title` - The title of the window.
  - ### Method Detail


    - #### combineFileNames

      ```
      protected final String combineFileNames(String file1,
                            String file2,
                            String ext)
      ```

      Combine two file names into a new file name.

      Parameters:
      :   `file1` - The first file name.
      :   `file2` - The second file name.
      :   `ext` - The extension of the new file name.

      Returns:
      :   The combined file name.


    - #### doActions

      ```
      protected void doActions(String command)
      ```

      **Description copied from class: `InternalWindow`**

      Do any actions specific to this window.

      **Specified by:**
      :   `doActions` in class `InternalWindow`

      Parameters:
      :   `command` - The command that signifies a particular action.


    - #### executeModuleAction

      ```
      protected abstract void executeModuleAction(String command)
      ```

      Execute an action within a particular module.

      Parameters:
      :   `command` - The command that signifies a particular action.


    - #### finishModule

      ```
      protected final void finishModule(String text)
      ```

      Handle finishing of a module.
        
        
      Finishing of a module involves closing of the window and showing any
      error that may have occurred.

      Parameters:
      :   `text` - The result of the calculation spawned by the module.


    - #### getInputControl

      ```
      protected final JComponent getInputControl(Container hold,
                               int index)
      ```

      Get a particular input control from a given container.

      Parameters:
      :   `hold` - The container that holds the input control.
      :   `index` - The index of the control to get, one-indexed.

      Returns:
      :   The input control.


    - #### getInputControl

      ```
      protected final JComponent getInputControl(int index)
      ```

      Get a particular input control from the content pane.

      Parameters:
      :   `index` - The index of the control to get, one-indexed.

      Returns:
      :   The input control.


    - #### makeInputControls

      ```
      protected abstract void makeInputControls()
      ```

      Make any input controls necessary to get data from this window for a
      calculation.


    - #### makeStartButton

      ```
      protected final void makeStartButton(int x,
                         int y)
      ```

      Create the "START" button.
        
        
      Note that this method simply makes the button, adds the action
      listener, applies standard padding, then places the button at the
      specified (X,Y) layout coordinate.
        
        
      If more customization of the button itself or its positioning is
      needed, it should be done in subclasses.

      Parameters:
      :   `x` - The X index at which to place the button in the layout.
      :   `y` - The Y index at which to place the button in the layout.


    - #### moduleInitialized

      ```
      protected final boolean moduleInitialized(String result)
      ```

      Check to see if the module was initialized correctly.
        
        
      Check what the result string retrieved from the module was, and return
      true only if it's the empty string. Otherwise, show the result string
      as an error and return false.

      Parameters:
      :   `result` - The result of the data structure construction.

      Returns:
      :   True if the data structure was initialized correctly,
          false if not.


    - #### placeComponent

      ```
      protected final void placeComponent(int x,
                        int y,
                        JComponent component)
      ```

      .
      Set a component in its place.

      Parameters:
      :   `x` - The X coordinate of the grid.
      :   `y` - The Y coordinate of the grid.
      :   `component` - The component to set in place.


    - #### replaceExtension

      ```
      protected final String replaceExtension(String file,
                            String extension)
      ```

      Replace the extension on a file name.

      Parameters:
      :   `file` - The file name whose extension to replace.
      :   `extension` - The new extension.

      Returns:
      :   The new file name with extension replaced.


    - #### runMainCalculation

      ```
      protected abstract void runMainCalculation()
      ```

      Run the module calculation in the back end.
        
        
      Data must be pulled from the module window before the back end
      calculation is run, so this data capture should be done in this method.


    - #### setAnchorCenter

      ```
      protected final void setAnchorCenter()
      ```

      Set the anchoring area for the GridBagConstraints to CENTER.


    - #### setAnchorNorth

      ```
      protected final void setAnchorNorth()
      ```

      Set the anchoring area for the GridBagConstraints to NORTH.


    - #### setFillCenter

      ```
      protected final void setFillCenter()
      ```

      Set the fill for the GridBagConstraints as CENTER.


    - #### setFillHorizontal

      ```
      protected final void setFillHorizontal()
      ```

      Set the fill for the GridBagConstraints as HORIZONTAL.


    - #### setGrid

      ```
      protected final void setGrid(int width,
                 int height)
      ```

      Set the amount of space a component takes up in the grid.

      Parameters:
      :   `width` - The width of the component.
      :   `height` - The height of the component.


    - #### setInsets

      ```
      protected final void setInsets(int top,
                   int left,
                   int bottom,
                   int right)
      ```

      Set the external padding of the constraints.

      Parameters:
      :   `top` - The top padding.
      :   `left` - The left padding.
      :   `bottom` - The bottom padding.
      :   `right` - The right padding.


    - #### setPad

      ```
      protected final void setPad(int xPad,
                int yPad)
      ```

      Set the internal padding of the constraints.

      Parameters:
      :   `xPad` - The padding in the X direction.
      :   `yPad` - The padding in the Y direction.


    - #### showProgressBar

      ```
      private void showProgressBar(boolean determinate)
      ```

      Create a progress bar.

      Parameters:
      :   `determinate` - True if the progress bar is determinate,
          false if not.


    - #### showProgressBarDeterminate

      ```
      protected void showProgressBarDeterminate()
      ```

      Create a determinate progress bar.


    - #### showProgressBarIndeterminate

      ```
      protected void showProgressBarIndeterminate()
      ```

      Create an indeterminate progress bar.


- Overview
- Package
- Class
- Tree
- Index
- Help

- Prev Class
- Next Class

- Frames
- No Frames

- All Classes

- Summary:
- Nested |
- Field |
- Constr |
- Method

- Detail:
- Field |
- Constr |
- Method
